# The proximity between styloid process and internal carotid artery as a possible risk factor for dissection: a case–control study

**DOI:** 10.1007/s00234-023-03121-0

**Published:** 2023-02-08

**Authors:** G. Venturini, L. Vuolo, G. Pracucci, A. Picchioni, Y. Failli, F. Benvenuti, C. Sarti

**Affiliations:** 1 Department of General Medicine, USL Toscana Centro, 50122 Florence, Italy; 2 Neuroradiology Unit, Careggi University Hospital, 50139 Florence, Italy; 3 NEUROFARBA Department, University of Florence, Viale Pieraccini 6, 50139 Florence, Italy; 4 Stroke Unit, Città Di Castello Hospital, USL Umbria 1, Via Luigi Angelini 10, Perugia, Italy; 5 Neurology Unit, Santo Stefano Hospital, USL Toscana Centro, Via Suor Niccolina Infermiera 20, 59100 Prato, Italy; 6 Health Science Department, Psychiatry Section, University of Florence, Viale Pieraccini 6, 50139 Florence, Italy

**Keywords:** Styloid process, Carotid artery dissection, Young adult, Stroke

## Abstract

**Purpose:**

The anatomical proximity of the styloid process (SP) to the ipsilateral internal carotid artery (ICA) has been recently recognized as a possible risk factor for carotid artery dissection (CAD). We aimed to verify this hypothesis by comparing the minimum distance between SP and ICA in young adult patients (< 55 years) with and without CAD.

**Methods:**

Thirty-one CAD patients (cases) were compared with 41 sex-matched patients without dissection, group one of control (G1), and with 16 sex-matched patients with vertebral artery dissection (VAD), group two of control (G2). Two independent observers measured, on CT angiography images, the minimum distance on the axial plane between the SP and ICA in cases and controls. They evaluated both the intercentric and the marginal distance. Differences between groups were estimated by Student *t*-test.

**Results:**

SP-ICA intercentric distance ipsilateral to dissection was significantly shorter compared to that of the contralateral side of cases (*p* < 0.001), to those of left and right side of G1 patients (*p* < 0.001 for both), and to those of left and right side of G2 patients (*p* < 0.001 for both). SP-ICA marginal distance of cases was significantly shorter compared to those of left and right side of G1 patients (*p* < 0.001 for both) and to those of left and right side of G2 patients (*p* < 0.001 for both).

**Conclusion:**

A short SP-ICA distance appears to be a risk factor for CAD as it likely induces a continuous microtraumatism of the vessel wall during normal head and neck movements.

## Introduction

Spontaneous cervical artery dissection (CeAD) is a common cause of ischemic stroke in young adults; according to the literature, it could be responsible for up to 20% of all events in people under the age of 55 years. Probably the true incidence is higher because many cases of dissection are undiagnosed due to mild or no symptoms [1, 2].

The anatomical classification of CeAD is based on the artery involved (carotid versus vertebral artery): the most common type is extracranial internal carotid dissection, which typically occurs 2–3 cm above the vessel bifurcation [3].

Its pathophysiology is only partially understood and seems to be multifactorial, due to the interaction between genetic, both monogenic (Ehlers-Danlos, Marfan, and Loyes-Dietz syndromes) and polymorphisms (C677T MTHFR gene, 844ins68bp CBS gene, G1958A MTHFD1 gene, E469K ICAM-1 gene, rs42524 COL1A2 gene, and others), and environmental factors such as minor cervical trauma (events associated with hyperextension or rotation of the neck including coughing, sneezing, vomiting, yoga, painting a ceiling, and chiropractic manipulation), infections, arterial tortuosity, hypertension, hypercholesterolemia, obesity [4–8]. The former predisposes the vessels to the dissection triggered by the latter.

**Fig. 1:**
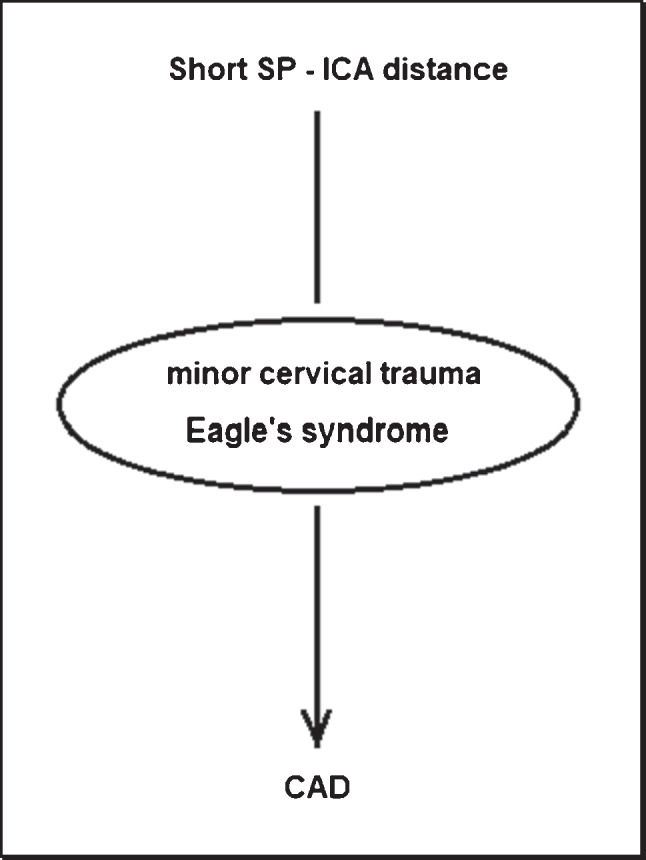
CAD etiology

Recent studies [9] suggest the existence of an association between the anatomical proximity of the styloid process (SP) and the risk of carotid artery dissection (CAD). SP is a pointed piece of bone that projects down and forward from the inferior surface of the petrous temporal bone to the ipsilateral internal carotid artery (ICA) and serves as an anchor point for several ligaments and muscles. This kind of relation could be also at the base of carotid artery dissection, which is the most feared complication of Eagle’s syndrome [10, 11], described for the first time in 1937 by Watt Weems Eagle, as a pain syndrome associated with an elongated SP. In both cases, the pathophysiological basis could be a microtraumatic mechanism of the vessel wall already predisposed by alterations of its connective tissues (Fig. 1).

The aim of our study is to confirm and possibly reinforce the preliminary literature data comparing the minimum distance between SP and ICA in young adult patients (< 55 years) with and without CAD. We decided to investigate only patients aged under 55 years to limit the effect of traditional cardiovascular risk factors in the determinism of CAD.

## Methods

We conducted a single-center retrospective case–control study. Each patient signed a written consent to the use of clinical data for research purposes, and the analysis (both statistical and radiological) was carried out on data previously anonymized by the patient management software.

### Selection and description of participants

Cases and controls were selected from patients, aged under 55 years, consecutively admitted to the stroke unit of our university hospital, from January 1, 2008, to August 31, 2019, for acute cerebrovascular events (TIA/ischemic stroke) for whom a CT-angiography (CTA) study of carotid and vertebral arteries on admission was available.

NIHSS score on admission and a history of vascular risk factors was collected for cases and controls.

**Fig. 2:**
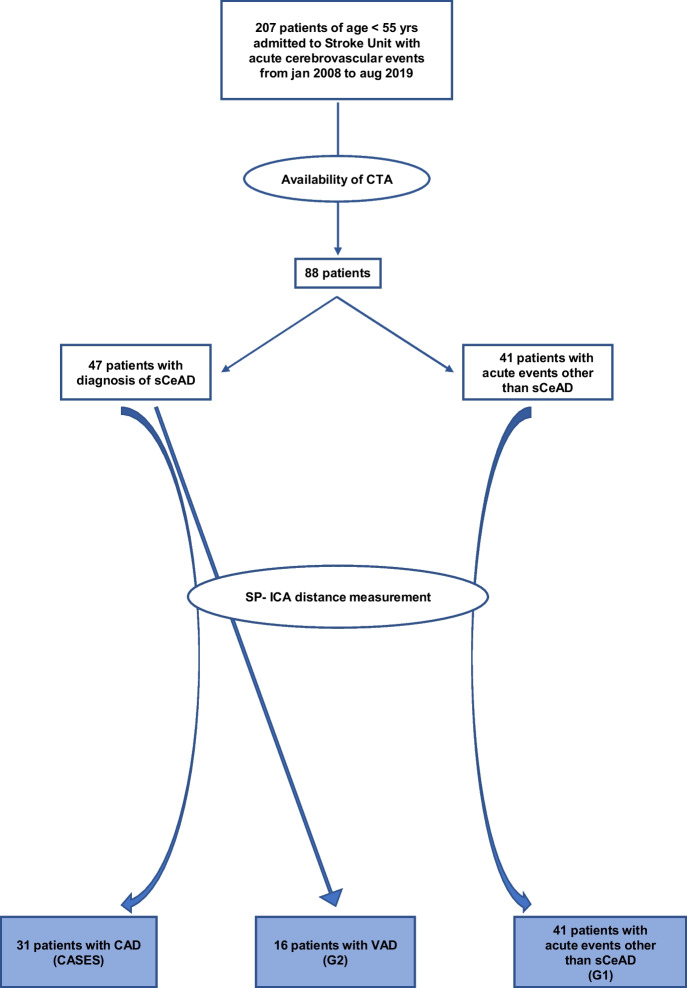
The flowchart of patient selection

From 207 patients aged under 55 years consecutively admitted to the stroke unit for acute cerebrovascular events (ACE), we selected 88 patients who underwent CTA on admission, 47 with and 41 without spontaneous CeAD (group one of control, G1). The 47 dissected patients were divided into 31 patients with CAD (group of cases) and 16 patients with VAD (group two of control, G2); we compared the SP-ICA distance of 31 CAD patients (cases) with that of 41 patients with ACE other than spontaneous CeAD (G1) and that of 16 VAD patients (G2) (Fig. 2).

### Technical information

Images were obtained with Siemens SOMATOM Definition AS + and Philips iCT SP scanners and reconstructed with Philips Intellispace Portal 7.0 software.

We used the angio acquisition package with minimal scan delay from reaching the ROI threshold (about 2.9 s delay); the reconstruction (standard kernel) was performed from the aortic arch to the cranial vertex: scan time 4 s = pitch 1.4 = rotation time 0.75 = modulation Z and 3D ON = KV 100 = mAs 251 (mAs range from 100 to 600), slice thickness 0.8 mm with an increment of 0.4 (overlapping images).

The contrast medium used was Iomeron 400 mg/mL in 60 mL with a flow equal to 5 mL/s.

**Fig. 3:**
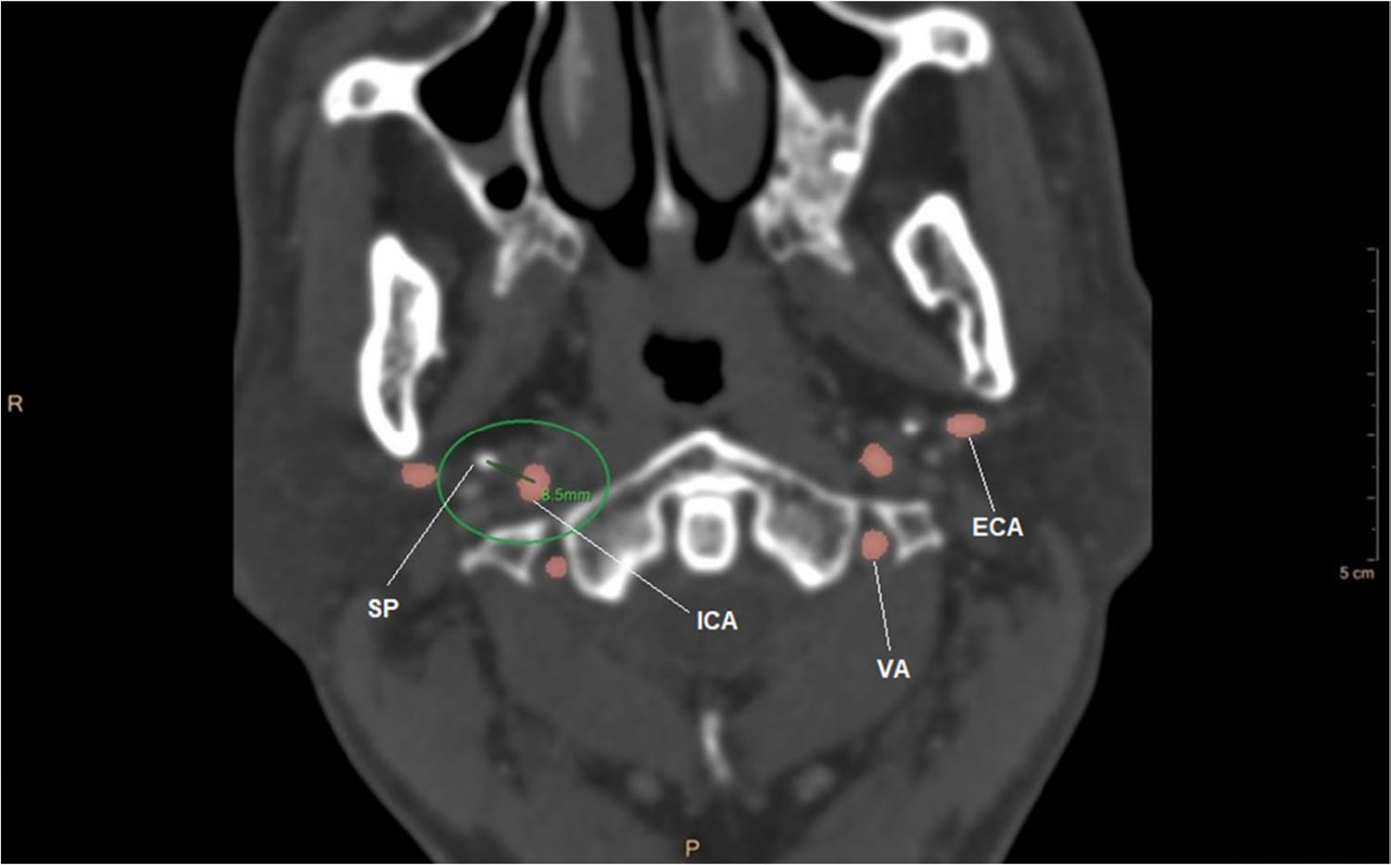
CT angiography image of intercentric distance (SP, styloid process; ICA, internal carotid artery; VA, vertebral artery; ECA, external carotid artery)
- the marginal distance, expressed in millimeters, from the edge of the vessel wall to that of SP (Fig. 4).

Two experienced neuroradiologists measured independently on axial projections of CTA images the minimum distance between the SP and ICA in cases and control groups. They evaluated:

- the intercentric distance, expressed in millimeters, from the geometric center of the vessel to that of SP (Fig. 3) and

**Fig. 4:**
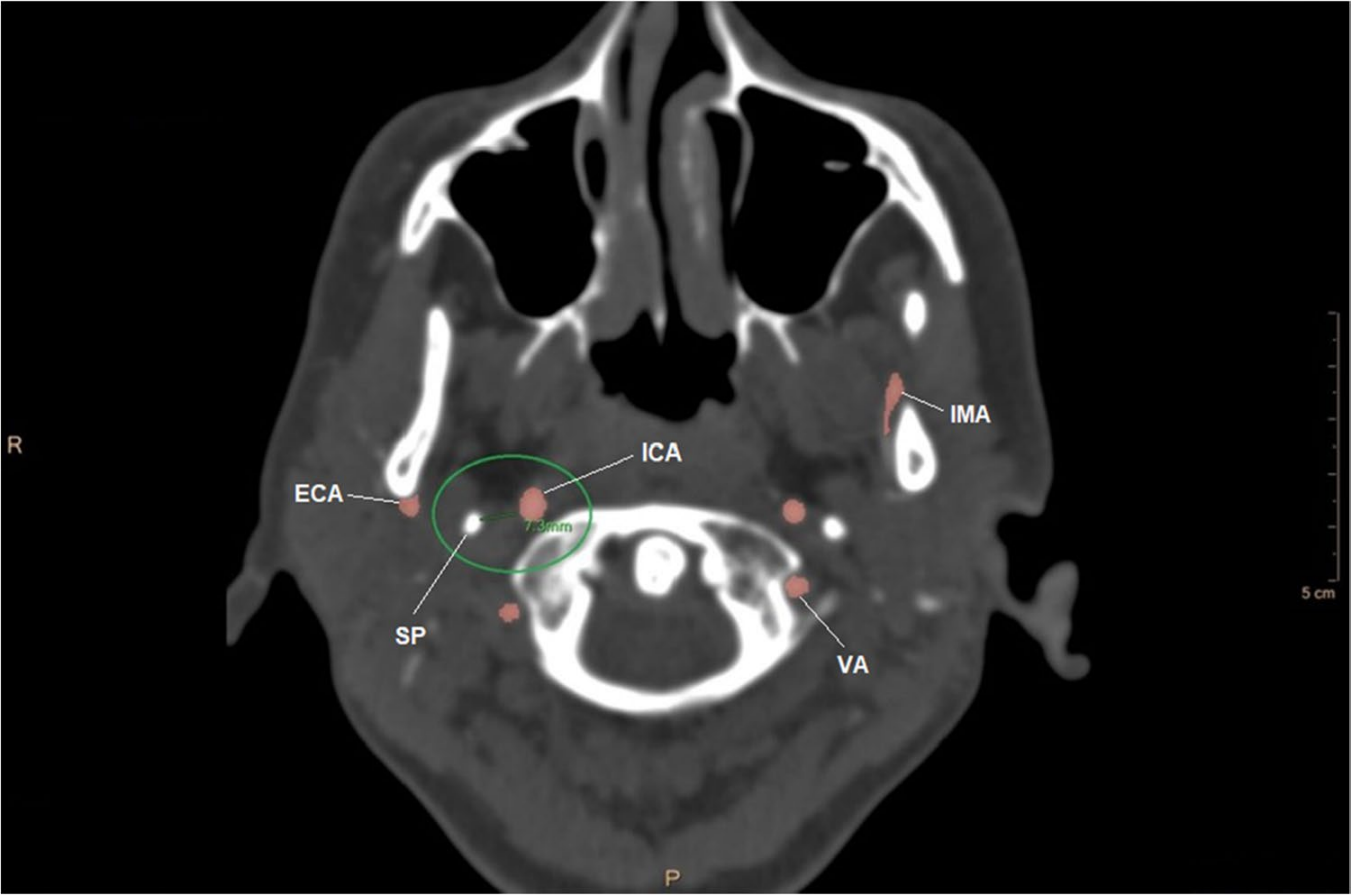
CT angiography image of marginal distance (SP, styloid process; ICA, internal carotid artery; VA, vertebral artery; IMA, internal maxillary artery)

We decided to include in the statistical analysis only the SP-ICA marginal distance contralateral to the dissection because the presence of the intramural hematoma at the site of dissection distorts the normal anatomical relationships, making the measurements less reliable.

### Statistics

All statistical analyses were performed using SPSS (Statistical Package for Social Sciences) IBM SPSS Statistics ver. 28. Differences between groups were analyzed by Student *t*-test, and Bland Altman analysis was used to measure the interrater agreement.

## Results

Concerning the distribution of the main epidemiological and clinical variables between cases (31) and G1 patients (41), the average age was higher in cases (47.09 ± 5.96 vs 42.85 ± 6.72), while the gender distribution was homogeneous (*p* = 0.059).

There was no significant difference between the two groups regarding the clinical severity of the disease measured on the basis of the NIHSS score (7.09 ± 7.30 in cases vs 8.00 ± 7.82 in G1, *p* = 0.744).

Minor cervical traumas were more frequent in patients with CAD, while dyslipidemia, active smoking, and moderate alcohol consumption (men: ≤ 3 units/day; women: ≤ 2 units/day) were significantly more represented in G1 patients (Table 1).

**Table 1:** Comparison of the epidemiological and clinical variables between cases and group one of controls (G1)

Variable	CAD *N* = 31 *x̄* ± SD or *p̂*	G1 *N* = 41 *x̄* ± SD or *p̂*	*p*
Age	47.09 ± 5.96	42.85 ± 6.72	0.007
Sex *m*	22/31 (71%)	20/41 (49%)	0.059
NIHSS	7.09 ± 7.30	8.00 ± 7.82	0.744
Sport	3/31 (10%)	2/39 (5%)	0.463
Minor trauma	6/18 (33%)	0/35 (0%)	< 0.001
Hypertension	11/31 (36%)	8/41 (20%)	0.128
Dyslipidemia	6/31 (19%)	17/25 (68%)	< 0.001
Obesity	2/11 (18%)	5/14 (36%)	0.332
Diabetes mellitus	0/31 (0%)	3/41 (7%)	0.124
Active smoking	6/31 (19%)	19/41 (46%)	0.017
Moderate alcohol consumption	6/31 (19%)	11/18 (61%)	0.003
Migraine without aura	6/31 (19%)	6/41 (15%)	0.595
Migraine with aura	2/31 (7%)	0/41 (0%)	0.099

*x̄*, mean; *p̂*, proportion

As regards the distribution of aforementioned variables between cases (31) and G2 patients (16), the average age was higher in cases (47.09 ± 5.96 vs 42.25 ± 5.94), and the gender distribution was homogeneous (*p* = 0.756). The clinical severity of the disease measured by NIHSS score was greater in cases than in G2 patients (7.09 ± 7.30 vs 3 ± 6.85). All the other variables are distributed evenly between the two groups (Table 2).

**Table 2:** Comparison of the epidemiological and clinical variables between cases and group two of controls (G2)

Variable	CAD *N* = 31 *x̄* ± SD or *p̂*	G2 *N* = 16 *x̄* ± SD or *p̂*	*p*
Age	47.09 ± 5.96	42.25 ± 5.94	0.01
Sex *m*	22/31 (71%)	11/16 (68%)	0.756
NIHSS	7.09 ± 7.30	3 ± 6.85	0.013
Sport	3/31 (9.7%)	1/16 (6.2%)	0.689
Minor trauma	6/18 (33%)	4/10 (40%)	0.724
Hypertension	11/31 (36%)	8/16 (50%)	0.336
Dyslipidemia	6/31 (19%)	3/16 (18.7%)	0.960
Obesity	2/11 (18%)	2/12 (16.6%)	0.923
Diabetes mellitus	0/31 (0%)	0/16 (0%)	n.s
Active smoking	6/31 (19%)	1/16 (6.2%)	0.231
Moderate alcohol consumption	6/31 (19%)	1/16 (6.2%)	0.231
Migraine without aura	6/31 (19%)	5/16 (31.25%)	0.361
Migraine with aura	2/31 (7%)	1/16 (6.2%)	0.978

*x̄*, mean; *p̂*, proportion; *n.s.*, not significant

**Fig. 5:**
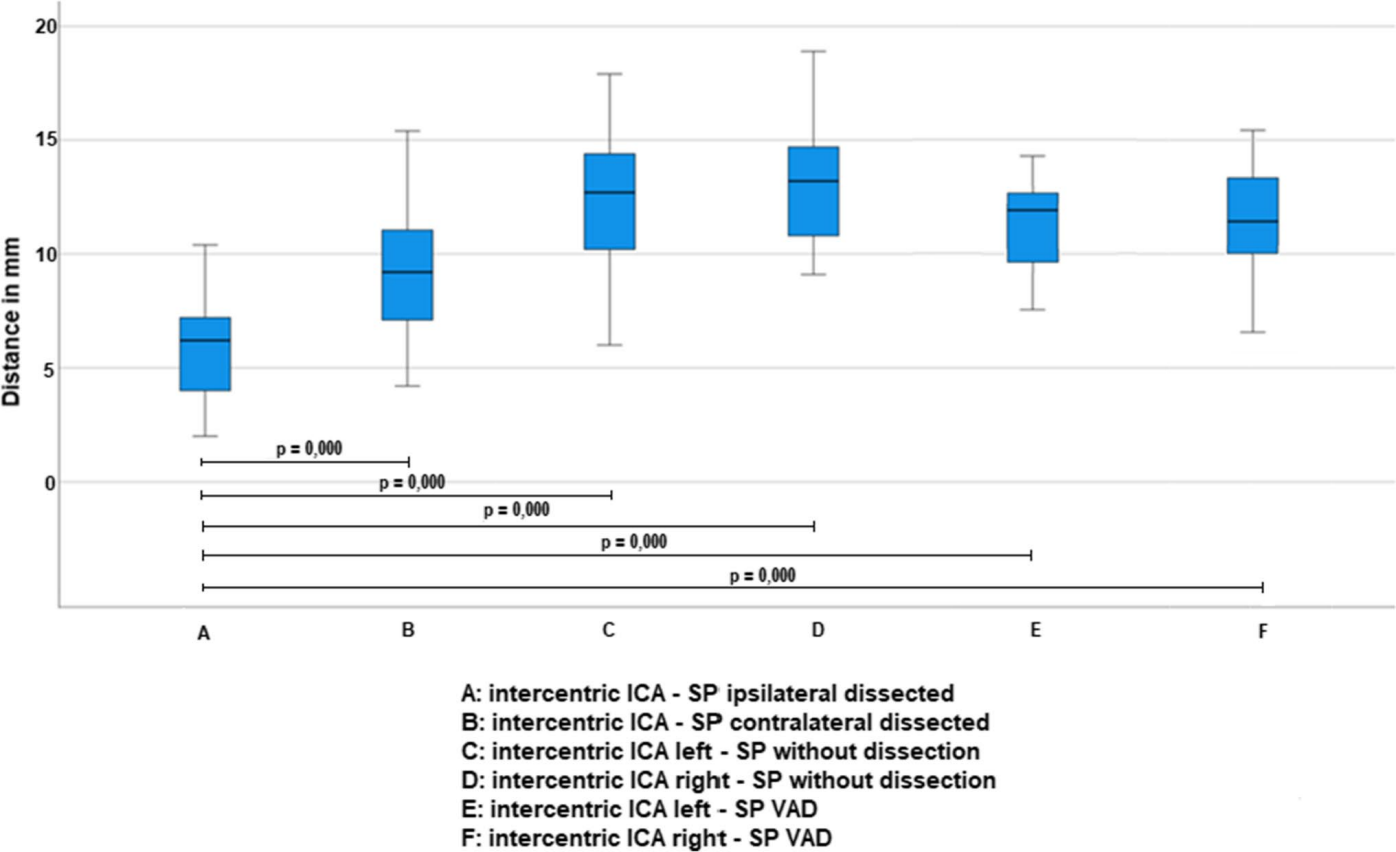
Comparison of intercentric distance between cases and controls

Concerning the distances on axial plane, SP-ICA intercentric distance ipsilateral to dissection was significantly shorter compared to that of the contralateral side of cases (5.9 ± 2.3 vs 9.3 ± 2.7; *p* < 0.001), to those of left (12.4 ± 2.9) and right side (13.0 ± 2.5) of G1 patients (*p* < 0.001 for both) and to those of left (12.0 ± 1.85) and right side (11.65 ± 2.3) of G2 patients (*p* < 0.001 for both) (Fig. 5).

**Fig. 6:**
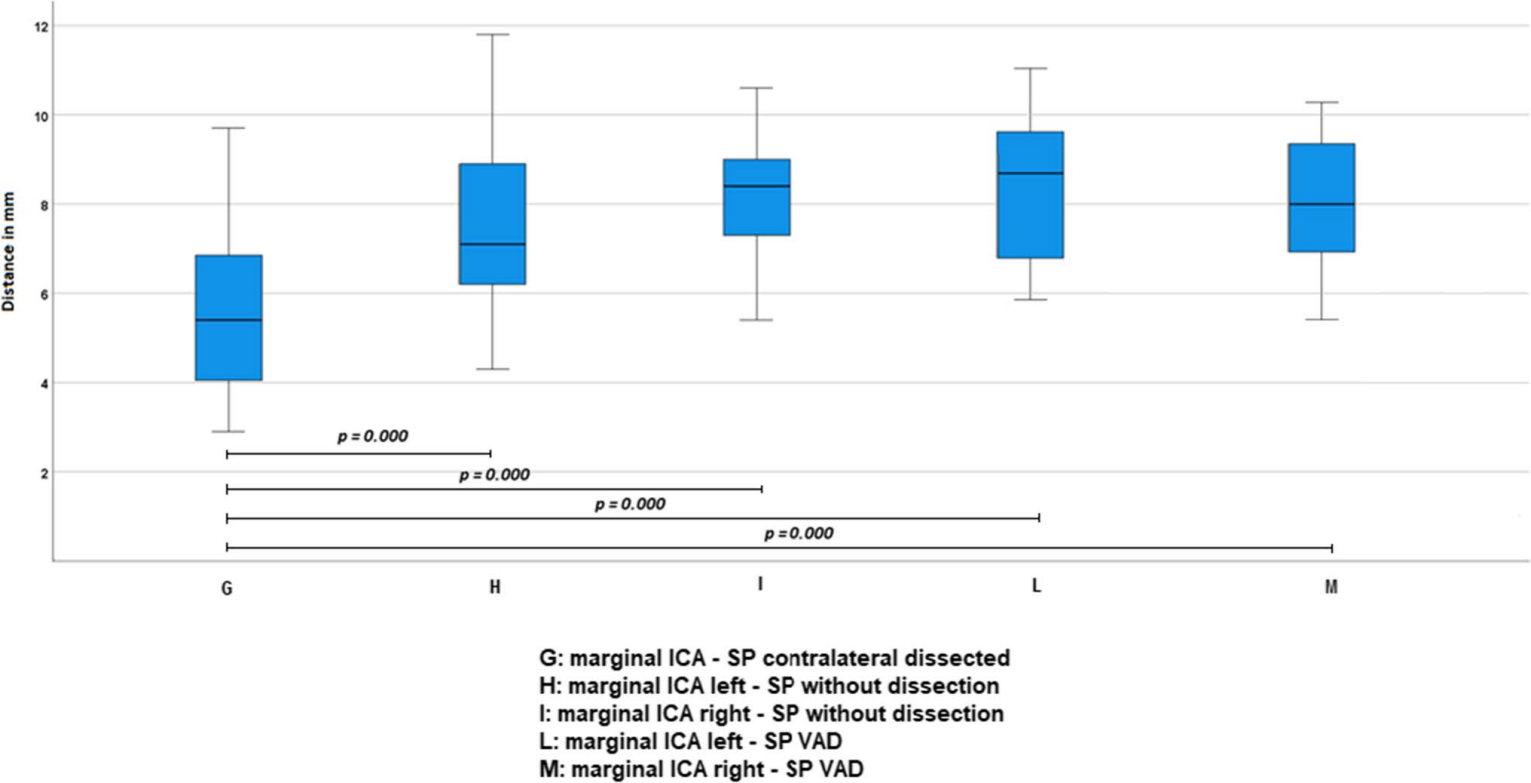
Comparison of marginal distance between cases and controls

SP-ICA marginal distance of cases (5.6 ± 2.1) was significantly shorter compared to those of left (7.5 ± 1.8) and right side (8.2 ± 1.3) of G1 patients (*p* < 0.001 for both) and to those of left (8.7 ± 1.71) and right side (8.05 ± 1.63) of G2 patients (*p* < 0.001 for both) (Fig. 6).

These results are confirmed also by pooling the patients of G1 and G2 in a single control group to be compared with that of cases (*p* < 0.001 for both intercentric and marginal distance).

**Fig. 7:**
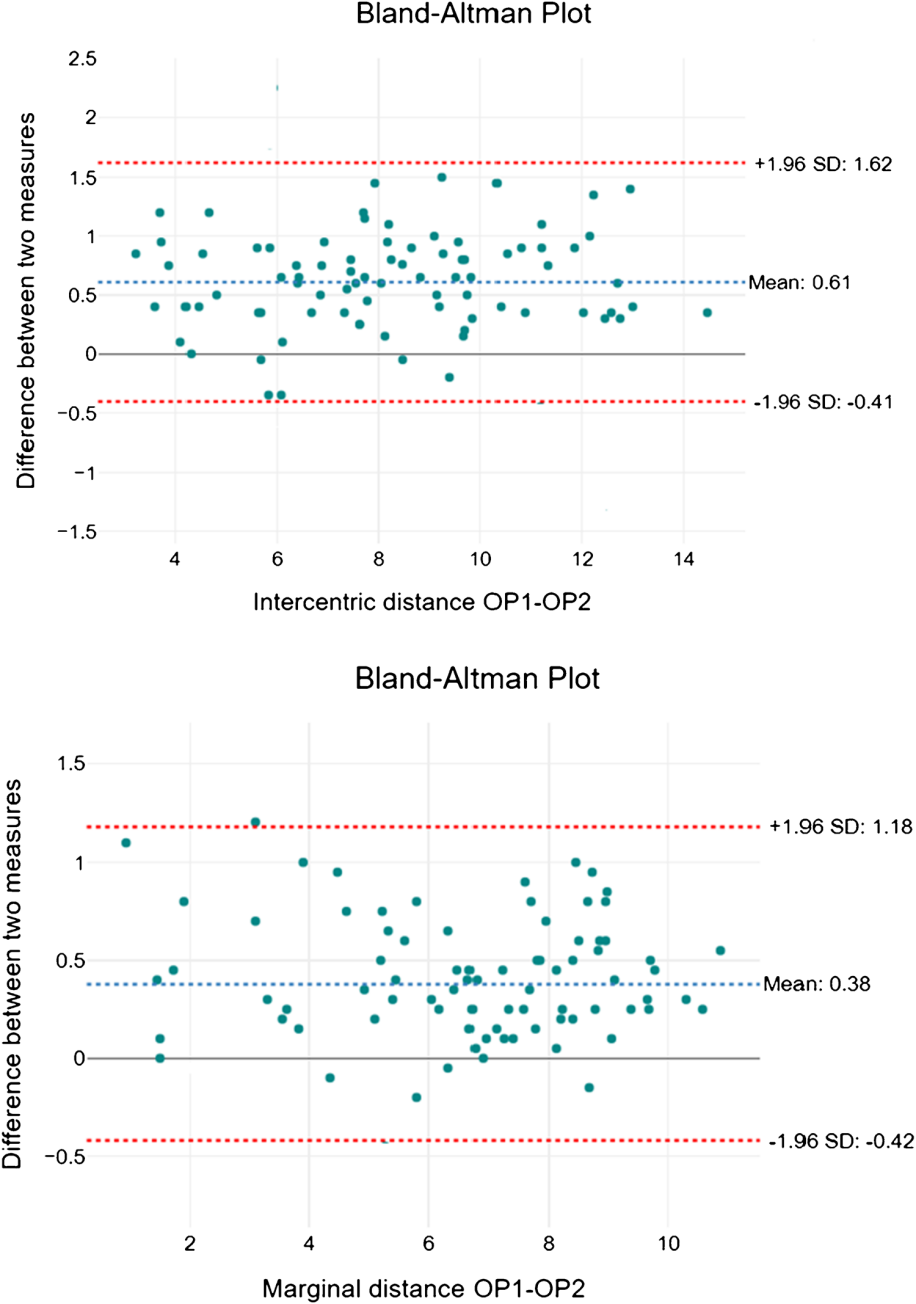
Bland Altman analysis: interrater agreement for intercentric and marginal distance (OP 1, operator 1; OP 2, operator 2)

Bland Altmann’s analysis revealed a good interrater agreement between the measurements independently taken by the two neuroradiologists: 95% of the differences between measurements are included within the limits of agreement (Fig. 7).

## Discussion and conclusions

Our study has confirmed data from previous literature suggesting that a short SP-ICA distance could be a risk factor for CAD.

Microtraumatism repeated over time on a vasal wall already weakened by minor, mainly unknown connective tissue alterations could be hypothesized to be the pathophysiological mechanism at the base of this association. Cervical extensions as well as sudden neck movements may compress the carotid artery against either the SP of the temporal bone or the transverse processes or the bony mass of the upper three cervical vertebrae, causing mechanical damage on vessels already predisposed.

Focusing on the methodology of our study, we decided to measure not only the marginal distance but also the intercentric one because intramural hematoma may distort ICA’s anatomy and its position in relation to other cervical structures. According to Amorim et al. [9], the distance between SP and the center of the structure formed by the hematoma and the arterial lumen is a parameter less influenced by the formation of intramural hematoma compared to the marginal distance. For the same reason, we decided not to include the SP-ICA marginal distance ipsilateral to the dissection in the statistical comparison.

We did not measure the length of the SP ipsilateral to carotid artery because, in our opinion, it is difficult to correlate with the dissecting event as the latter could be also influenced by the position of SP in the space with respect to the vessel; in fact, if SP elongation is bilateral in most cases, bilateral CAD is not frequent.

In addition to these considerations, many predisposing factors could participate in the realization of CAD and the occurrence of dissection at a specific time and in a specific patient could derive from the combination of several contributors.

We also carried out a comparative evaluation between the SP-ICA distance of cases and that of patients with VAD (G2), reaching the same results as that with patients without dissection (G1): this could support the hypothesis that carotid and vertebral arteries should be considered two independent districts also assuming the presence of an underlying condition characterized by an alteration of the vascular connective tissue [5].

The main limitations of our study are the following:

1. the case–control design conducted on symptomatic patients: the ideal study to answer the question of anatomical proximity between SP and ICA may represent a risk factor for spontaneous CAD should be a longitudinal observational study conducted in the general population of young adults, even asymptomatic, but appears difficult to achieve.

Indeed, symptomatic patients could represent a very specific population group because received medical attention and detailed neuroimaging studies that could detect a short SP-ICA distance, which could be just an epiphenomenon of an unknown pathophysiological mechanism or just a small component of a very complex multifactorial process;

2) the single-center design: this could have introduced a patient selection bias;
3) the quite small number of patients in the three groups may have underpowered our results;
4) the loss of a part of patients in the selection process of cases and controls may have introduced a selection bias in all groups;
5) the lack of a standardized method to identify the edge and the center of both the carotid vessel and the SP by neuroradiologists on axial projections could have introduced inaccuracy in the measurements. This potential bias is reduced by the presence of two radiologists independently performing the measurements.

With these limitations, this study opens up new insights and stimulates further explorations of the still largely unknown pathogenesis of this frequent and potentially very severe disease that primarily affects young adults.

On a practical level, the SP-ICA distance could be used as a part of a composite index (together with other factors including age, sex, vascular tortuosity, dyslipidemia, active smoking, cervical manipulations, etc.) which estimates the risk of carotid dissection in all those patients who, regardless of the cause, undergo to a CT- or a MR-angiography. This could allow clinicians to intervene on modifiable factors and/or recommend any primary prevention therapy (pharmacological or, in very selected cases, surgical).
